# A Rare Case of a Child Diagnosed With Multisystem Inflammatory Syndrome After COVID-19 Presenting With Renal Infarctions and Transient Blast Cells: A Case Report and Literature Review

**DOI:** 10.7759/cureus.30832

**Published:** 2022-10-29

**Authors:** Mohammed A Almatrafi, Abdulrahman F Kabli, Yara Subahi, Esraa Yaseen, Nouf Alsahaf, Dhuha Alidrisi, Hanan A Ahmed, Hassan M Masmali, Ossamah Alahmad, Mohammad N Khan, Faisal Minshawi

**Affiliations:** 1 Department of Pediatrics, Umm Al-Qura University, Makkah, SAU; 2 Department of Medicine, Umm Al-Qura University, Makkah, SAU; 3 Department of Pediatrics, Security Forces Hospital, Makkah, SAU; 4 Department of Pediatrics, Maternity and Children Hospital, Makkah, SAU; 5 Department of Laboratory Medicine, Umm Al-Qura University, Makkah, SAU

**Keywords:** blast cell, acute renal injury, pediatric inflammatory multisystem syndrome, sars-cov-2 infection, covid-19 virus infection

## Abstract

Multisystem inflammatory syndrome in children (MIS-C) can develop weeks after the Coronavirus disease 2019 (COVID-19). The disease’s clinical spectrum includes persistent febrile illness, features resembling Kawasaki disease, and cytokine storm symptoms. In severe cases, multisystem organ failure and death may result if not treated promptly. This report discusses a rare case of a 13-year-old girl presenting with fever and acute kidney injury (AKI) eight weeks after recovering from COVID-19 who was diagnosed with MIS-C.

A 13-year-old female presented with a fever and abdominal pain following a recent COVID-19. A physical examination revealed a febrile, ill-looking child with abdominal tenderness. Pancytopenia, transaminitis, AKI, and a hyperinflammatory state were noted in the initial laboratory workup. Furthermore, blast cells were seen on the peripheral blood smear. Despite appropriate empiric antibiotic therapy for sepsis, she did not show signs of clinical improvement. An abdominal computed tomography (CT) scan revealed multiple focal areas of hypoattenuating lesions involving both kidneys, suggestive of bilateral renal infarction. Since she met the criteria of the Centers for Disease Control and Prevention (CDC) for MIS-C diagnosis, a high dose of intravenous immunoglobulin (IVIG) led to a dramatic improvement in the patient’s condition and complete recovery from her illness.

This case report describes a rare clinical presentation of MIS-C in a child who presented with AKI due to presumably thrombotic events and transient blast cells in blood film secondary to a severe inflammatory process. Further studies are needed to determine the prevalence of thrombotic AKI associated with MIS-C.

## Introduction

COVID-19 was discovered in December 2019 in Wuhan, China, and is caused by the severe acute respiratory syndrome coronavirus 2 (SARS-CoV-2) [1]. SARS-CoV-2 is a highly contagious viral respiratory illness that affects individuals of all ages [2]. Although it is most often associated with the respiratory system, it may also affect other systems [2]. The Centers for Disease Control and Prevention (CDC) in the United States issued a warning in May 2020, identifying a novel condition known as a multisystem inflammatory syndrome in children (MIS-C) associated with COVID-19 [3]. MIS-C symptoms may manifest several weeks after COVID-19 is contracted [3]. Clinically severe febrile illness requiring hospitalization in children younger than 21 years of age with elevated markers of inflammation and involvement of at least two organ systems with no apparent plausible explanation in the context of recently confirmed or suspected COVID-19 would fulfill the CDC case definition of MIS-C in children [3].

Depending on the severity, patients with MIS-C may develop acute renal, cardiac, or liver injuries [3,4]. Lipton et al. reported that 46% of pediatric patients diagnosed with MIS-C had acute kidney injury (AKI), but all of these patients had favorable outcomes and recovered completely before hospital discharge [4]. It has been reported that coagulopathy associated with MIS-C puts children at a higher risk of developing thrombotic events than patients with milder forms of COVID-19 [5]. Hematological abnormalities with stressed bone marrow (BM) have also been reported in patients with MIS-C [6,7]. Anemia, leukopenia, lymphopenia, neutrophilia, and thrombocytopenia have all been described [3]. This report describes a rare case of a 13-year-old girl diagnosed with MIS-C who presented with fever and AKI eight weeks after recovering from COVID-19.

## Case presentation

A previously healthy 13-year-old female was admitted with a history of high-grade fevers, generalized abdominal pain, and non-bilious, non-bloody emesis for three days. She sought medical attention at a local pediatric emergency department and was discharged home with supportive care for possible viral gastroenteritis. Eight weeks prior to hospital admission, the patient and her family members were diagnosed with COVID-19 using the Xpert Xpress SARS-CoV-2 test, powered by Cepheid Innovation (Sunnyvale, CA), from nasopharyngeal swab specimens. She was fully immunized with routine vaccines but had not received COVID-19 immunization at the time of presentation.

At presentation, the patient was in moderate pain and ill-looking. Vital signs were noted for fever (39.5°C), marked tachycardia (140 beats per minute), and tachypnea (30 breaths per minute). While she maintained her blood pressure, it was at the lower end of the normal range for her age (94/50). The initial abdominal examination revealed diffuse abdominal tenderness without visceral enlargement or palpable masses, but the tenderness became more confined to her costovertebral angles bilaterally during the following days. The remainder of her exam was unremarkable.

Her initial laboratory workup revealed pancytopenia in her complete blood count. She had leukopenia (white blood cell count: 2041 cells/mm^3^), lymphopenia (lymphocytes: 326 cells/mm^3^, 16%), anemia (hemoglobin: 10.4 g/dL) with increased reticulocyte count (4.5%), and thrombocytopenia (platelet count: 113,000/mm^3^). She had AKI, as evidenced by an elevated serum creatinine level from her baseline (0.8 mg/dL, patient’s baseline 0.35 mg/dL). The liver function tests revealed mild transaminitis (alanine transferase 96 U/L; aminotransferase 61 U/L) and a low albumin level (29 g/L). Although partial thromboplastin time was elevated (44.3 seconds), prothrombin time and international normalized ratio (INR) were normal. She was in a hyperinflammatory state with elevated C-reactive protein (CRP) (19 mg/dL), ferritin (783 μg/L), lactate dehydrogenase (286 U/L), and D-dimer (4 mg/L). Urinalysis showed non-nephrotic range proteinuria and microscopic hematuria with no pyuria. Rheumatological workup, including antinuclear antibodies, double-stranded DNA antibodies, and complement levels, was normal. Two SARS-CoV-2 polymerase chain reactions from nasopharyngeal specimens performed while the patient was hospitalized returned negative, indicating that reinfection with SARS-CoV-2 was unlikely. Ceftriaxone (80 mg/kg/day) and vancomycin (60 mg/kg/day) were started for sepsis during the wait for blood and urine culture results.

**Figure 1:**
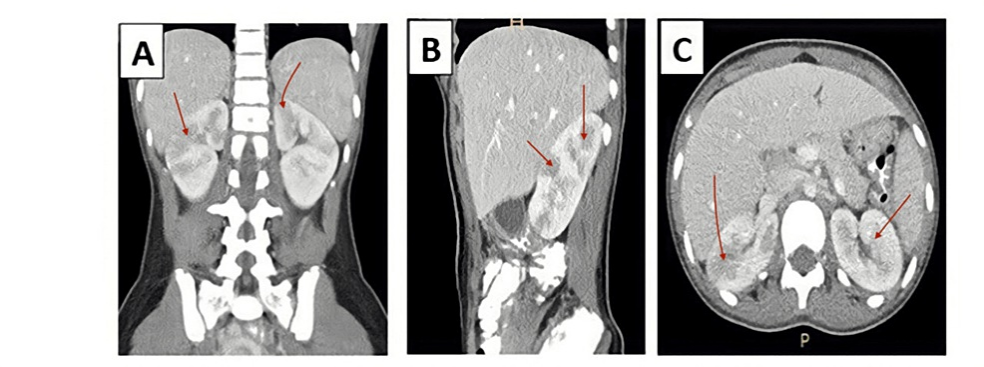
Coronal (A), sagittal (B), and axial (C) views of abdominal computed tomography scan with intravenous contrast. The arrows indicate multiple focal areas of hypoattenuating lesions involving both kidneys, suggestive of bilateral renal infarcts.

A computed tomography (CT) scan of the abdomen with intravenous contrast was obtained due to continued significant costovertebral angle abdominal pain and tenderness. Multiple focal areas of hypoattenuating wedge-shaped lesions involving both kidneys were seen on abdominal imaging, raising suspicion of renal infarctions (Figure 1).

**Figure 2:**
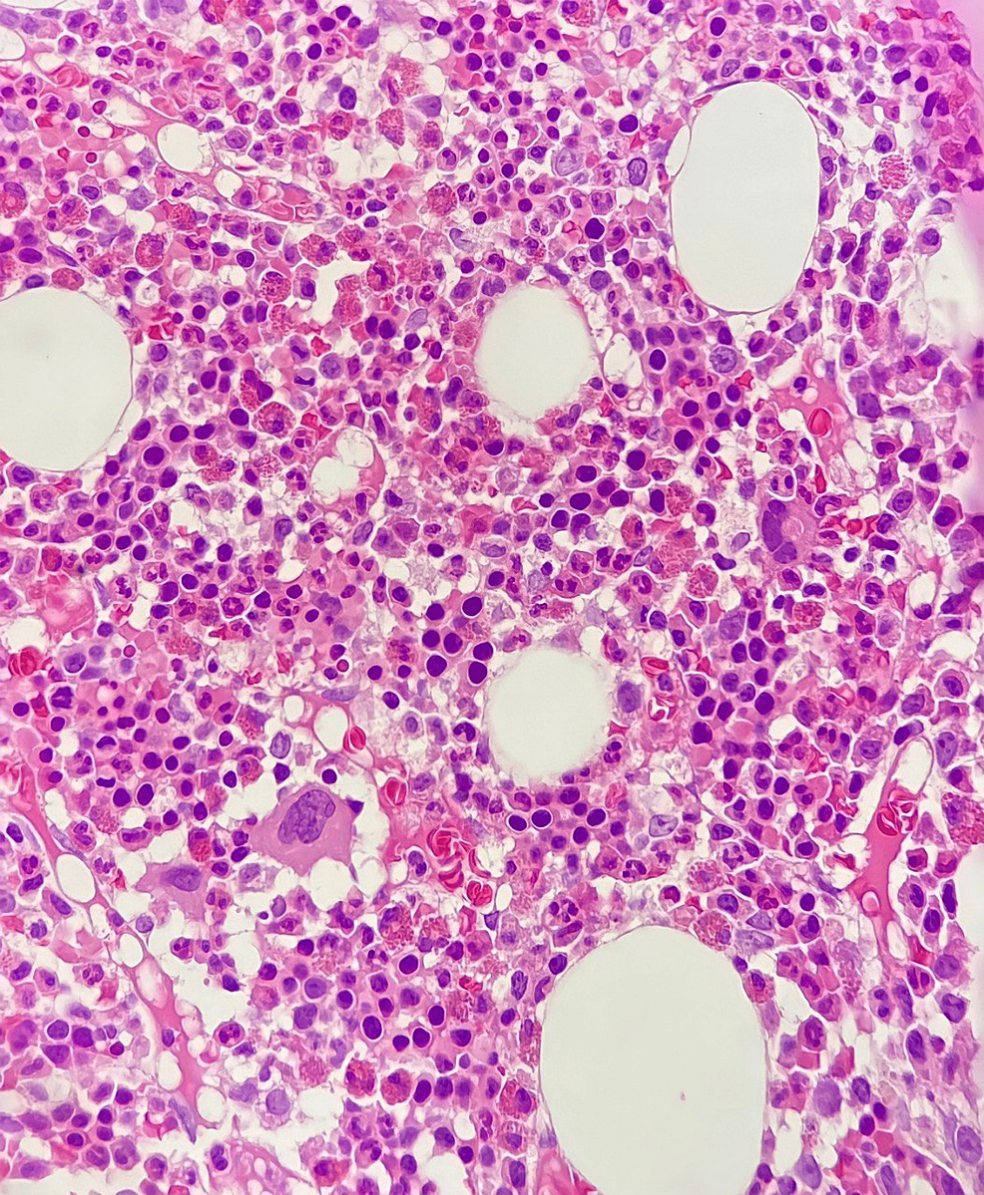
Histopathology of the bone marrow sample. Histopathology of the bone marrow revealed a normocellular specimen, active trilineage hematopoiesis, and no evidence of bone marrow infiltration by neoplastic cells.

On the blood film, there were neutropenia, normoblasts, and atypical lymphocytes. Flow cytometry revealed 4% blast cells with CD45dim and CD34+ expression, which raised suspicions of acute myeloid leukemia; therefore, a BM biopsy was performed. The biopsy revealed a normocellular BM, active trilineage hematopoiesis, and no evidence of BM infiltration by neoplastic cells (Figure 2). Persistent fevers and worsening clinical status with labile blood pressure despite broad-spectrum antibiotics increased suspicion of MIS-C, especially with the recent history of COVID-19 infection. Additionally, blood and urine cultures came back negative. In our patient, the presence of severe symptoms requiring hospitalization, involvement of more than two systems with AKI, hematological abnormalities, mild liver injury, the presence of a hyperinflammatory state (elevated CRP, low albumin, high ferritin, and high D-dimer), and reported recent COVID-19 in the absence of an alternative explanation for her current illness fulfill the diagnostic criteria of MIS-C. Although the average time between acute COVID-19 and the onset of MIS-C is two to six weeks, rare cases of MIS-C occurring after six weeks have been described in the medical literature [8]. Therefore, a high dose of intravenous immunoglobulin (IVIG) was administered (2 mg/kg, maximum dose 70 mg). A dramatic improvement in the patient’s clinical status and laboratory results was observed after the IVIG infusion was started.

**Figure 3:**
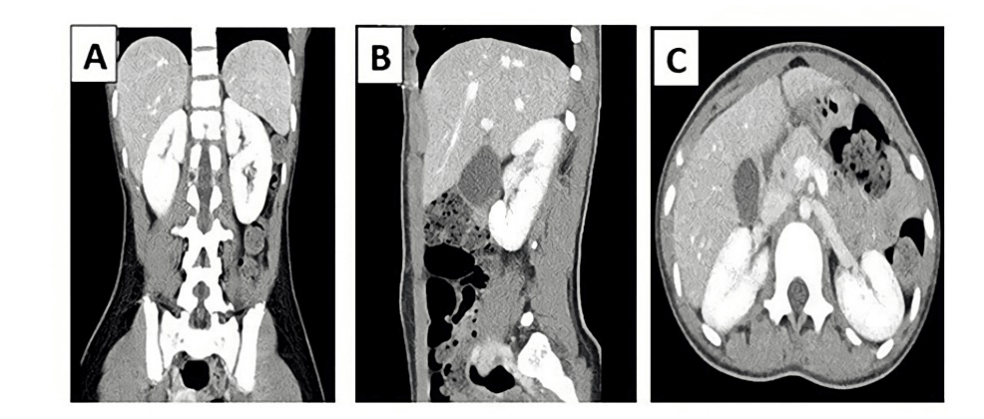
Coronal (A), sagittal (B), and axial (C) views of repeat abdominal computed tomography scan with intravenous contrast. Coronal, sagittal, and axial views of repeat abdominal CT scan show complete resolution of renal lesions following successful treatment of MIS-C.

The patient was discharged home in good health condition. After one week, repeat tests showed complete resolution of all inflammatory parameters and renal lesions (Figure 3).

## Discussion

This report describes a rare clinical presentation of MIS-C in a child with transient blast cells and AKI presumably due to thrombotic emboli. MIS-C is believed to result from an abnormal immune response to COVID-19 [6,9]. Although the exact mechanism is unknown, a postinfectious process is hypothesized based on the timing of these cases in relation to the peak of COVID-19 cases in communities [6,9]. While MIS-C shares some clinical characteristics with cytokine release syndrome, Kawasaki disease, and macrophage activation syndrome, the epidemiology of these other conditions is quite different [6,9]. Most MIS-C cases have occurred in previously healthy older children and adolescents [10,11], whereas Kawasaki disease, for example, typically affects younger children and infants, with a higher incidence in children of Asian ancestry [12,13].

Our patient met the CDC diagnostic criteria for MIS-C, but the presence of bilateral renal lesions that are suggestive of renal infarcts, in addition to blast cells on the peripheral blood smear, was extremely unusual. Acute renal infarctions are uncommon in the pediatric age group and are frequently associated with underlying pathologies such as cardiac arrhythmias, valvular diseases, infective endocarditis, hypercoagulable states, and dissection of the renal arteries [14,15]. Whitworth et al. found that patients with MIS-C had a greater rate of thromboembolic events than those with mild or asymptomatic COVID-19 infections (6.5% vs. 2.1% vs. 0.7%, respectively) [5].

COVID-19 infection was recently identified as a possible cause of renal infarctions in published case reports and series [16,17]. Plouffe et al. described a pediatric patient who developed fever, abdominal pain, and bilateral renal infarcts due to thrombotic events triggered by MIS-C [18]. Our patient presented similarly, with the caveat of transient blast cells on the blood smear. The history of recent COVID-19 eight weeks before hospital admission and the lack of any identifiable risk factors for her AKI secondary to renal infarcts lead us to suspect that MIS-C also triggered her hyperinflammatory state and renal lesions. Both cases had favorable outcomes, with complete resolution on follow-up [18]. The patient reported by Plouffe et al. was treated with ceftriaxone for a few days before being discharged on aspirin 81 mg for six months. Comparatively, our patient responded exceptionally well to IVIG, as evidenced by a remarkable improvement in her clinical condition, normalized laboratory findings, and complete resolution of renal lesions.

The presence of blast cells in our patient’s peripheral blood was one of the most striking findings in her laboratory results. Although the blast cell percentage was only 4%, the expression of CD45dim and CD34+ markers in flow cytometry analysis raised suspicions of acute myeloid leukemia. The low blast cell count on the peripheral blood smear, active BM demonstrated by an increased reticulocyte count, and the patient’s rapid recovery all argued against that suspicion. Fortunately, no evidence of a neoplastic process was detected in her BM biopsy. Blast cells can be seen in the peripheral blood of patients who have developed a leukemoid reaction as a result of sepsis or who have received granulocyte colony-stimulating factor therapy [7,19]. Aladily et al. described a transient increase in blast count of 6% on the peripheral blood smear and 23% in the BM of a 54-year-old COVID-19 patient, mimicking an acute leukemia case [7]. However, blood cells gradually decreased to normal levels without specific therapy, with normal BM cellularity and the absence of blast cells seen on subsequent BM biopsies [7]. Similar findings were seen in our patient, with significant improvements following the initiation of IVIG infusion. On follow up, repeated blood tests and CT scans showed complete normalization of all inflammatory markers and resolution of renal lesions.

## Conclusions

MIS-C has varied clinical presentations in children. Coagulopathy associated with MIS-C increases the risk of thromboembolic events in children. This report describes a rare clinical presentation of MIS-C in a child who presented with AKI due to thrombotic events and transient blast cells on blood film secondary to a severe inflammatory process. Clinicians should consider MIS-C in the differential diagnosis of patients recently exposed to or diagnosed with COVID-19 and presenting with high inflammatory states and thromboembolic events. By presenting this case, we aim to increase physicians' awareness of the various complications associated with MIS-C. Further studies are required to determine the prevalence of thrombotic AKI associated with MIS-C.
